# Design of a Novel Recombinant Multi-Epitope Vaccine against Triple-Negative Breast Cancer

**DOI:** 10.52547/ibj.26.2.160

**Published:** 2022-01-29

**Authors:** Hassan Dariushnejad, Vajihe Ghorbanzadeh, Soheila Akbari, Pejman Hashemzadeh

**Affiliations:** 1 Department of Medical Biotechnology, Faculty of Medicine, Lorestan University of Medical Sciences, Khorramabad, Iran;; 2 Razi Herbal Medicines Research Center, Lorestan University of Medical Sciences, Khorramabad, Iran;; 3 Department of Obstetrics and Gynecology, Lorestan University of Medical Sciences, Khorramabad, Iran

**Keywords:** Adjuvants, Triple-negative breast cancer, Vaccine

## Abstract

**Background:**

TNBC is determined by the absence of ERBB2, estrogen and progesterone receptors’ expression. Cancer vaccines, as the novel immunotherapy strategies, have emerged as promising tools for treating the advanced stage of TNBC. The aim of this study was to evaluate CEA, MTDH, and MUC-1 proteins as vaccine candidates against TNBC.

**Methods:**

In this research, a novel vaccine was designed against TNBC by using different immunoinformatics and bioinformatics approaches. Effective immunodominant epitopes were chosen from three antigenic proteins, namely CEA, MTDH, and MUC-1. Recombinant TLR4 agonists were utilized as an adjuvant to stimulate immune responses. Following the selection of antigens and adjuvants, appropriate linkers were chosen to generate the final recombinant protein. To achieve an excellent 3D model, the best predicted 3D model was required to be refined and validated. To demonstrate whether the vaccine/TLR4 complex is stable or not, we performed docking analysis and dynamic molecular simulation.

**Result:**

Immunoinformatics and bioinformatics evaluations of the designed construct demonstrated that this vaccine candidate could effectively be used as a therapeutic armament against TNBC.

**Conclusion:**

Bioinformatics studies revealed that the designed vaccine has an acceptable quality. Investigating the effectiveness of this vaccine can be confirmed by supplementary *in vitro* and *in vivo* studies.

## INTRODUCTION

Breast cancer disease, the utmost diagnosed malignant tumor in women, has been known as a major cause of female death^[^^1^^]^. Among the various subtypes of breast cancer, TNBC is an aggressive form of invasive and metastatic breast cancer. This subtype is determined by the absence expression of PR, ER, and human epidermal growth factor receptor type 2^[^^2^^]^. The overall outcome of TNBC tumors is poor, mainly due to the lack of effective targeted treatments for this type of cancer. TNBCs do not respond to currently available targeted treatments such as Herceptin or endocrine therapy^[^^3^^]^. On the other hand, TNBC tends to be aggressive, and at present, the cytotoxic chemotherapy is the only treatment option. For these reasons, new therapies are an urgent need for the treatment of this unmet medical advanced malignancy^[^^2^^,^^4^^]^.

Among the various novel treatments that have been introduced in recent decades, subunit vaccines that control cancer cells are considered as the most attractive armament against this malignancy^[^^5^^]^. The novel therapeutic vaccines are the next vaccine generation for the exploitation of the immune system against cancer. Antigen, epitope, adjuvant, and linker selection are critical factors for multi-epitope vaccine design, which can affect the clinical outcomes^[^^6^^]^. Nowadays, bioinformatics reduces the cost, risks, and time of drug design by introducing the best antigens and identifying potential epitopes. Immunoinformatic, as an attractive branch of bioinformatics, can assist biologists to predict immunogenic epitopes of target antigens^[^^7^^]^. 

Novel cancer vaccines usually consist of T-cell and B-cell epitopes from tumor-associated antigens or tumor-specific antigens^[^^8^^]^. The first step in designing a multi-epitope vaccine is to find suitable antigens that are overexpressed in breast cancer. After reviewing the literature, three antigens were selected, and used for vaccine design. The first selected antigen was CEA, a glycoprotein with a molecular weight of 180 kDa. This antigen has been shown to be overexpressed in most types of cancers, including gastric, colorectal, non-small cell lung, pancreatic, and breast cancers. CEA is an adhesion molecule and its overexpression in tumor cells caused metastasis^[^^9^^]^. The second selected antigen was MTDH, an important protein overexpressed in different types of cancers, including esophageal squamous cell carcinoma and prostate, liver, and breast cancers. Previous studies have shown that the amino acid sequence of 378 to 440 of this antigen is responsible for metastasis and migration of breast cancer tumor cells to the lung^[^^10^^]^. The last chosen antigen was MUC-1, a glycoprotein expressed on the surface of many types of malignant ductal epithelial cells, including breast, gastrointestinal tract, pancreas, and lung. This antigen overexpressed in approximately 70% of malignant cells and was sufficiently immunogenic to induce strong antitumor immune response as a tumor-associated antigens. These reasons make this antigen potential target for vaccine design^[^^11^^]^. 

Despite the certain benefits of multi-epitope vaccines, the weak immunogenicity is one of the main drawbacks for their clinical applications. To overcome this problem and enhance the protective immunity, additional components termed adjuvants can be added to strengthen the T-cell and B-cell immune responses^[^^5^^]^. Using TLR agonists as adjutant is one of the strategies for immune response enhancement. These agonists are derived from different types of microbes. It has been proved that TLR4 agonist from *Mycobacterium tuberculosis*, among other known TLR agonists, can be used as a strong adjuvant to multi-epitope cancer vaccines. TLR4 agonist has strong immune effects on tumors and can be applied as an adjuvant in the treatment of cancer^[^^12^^]^. In the present study, we introduce a multi-epitope vaccine against TNBC using immunoinformatics approaches. To achieve the desired results, we focused on three important antigens, namely MUC-1, CEA, and MTDH. In addition, TLR-4 agonist was added to the novel vaccine construct as an adjutant for stimulating the immune system.

## MATERIALS AND METHODS

**Multi-epitope vaccine design**

**Fig. 1:**
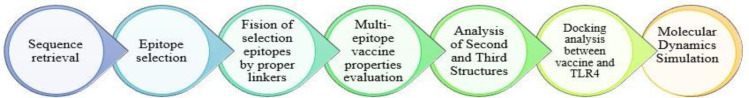
Schematic procedure algorithm for designing the novel multi-epitope vaccine. This schematic procedure briefly describes designing the multi-epitope vaccine

Figure 1 shows the schematic methodology and overall procedures used in this research.

**Retrieval of CEA, MTDH, MUC-1, and adjuvants sequence**

The complete amino acid sequence of three breast cancer-associated antigens were retrieved from The UniProt database (https://www.uniprot.org)^[^^13^^]^. These antigens include CEA (accession no. P06731), MTDH (accession no. Q86UE4), MUC-1 (accession no. P15941). The putative adjuvants, RpfB (accession no. P9WG29), and RpfE (accession no. O53177) were also selected in FASTA format. The PDB database (https://www.rcsb.org/pdb/home/home.do) was also used for the retrieval of the TLR4/MD2 complex structure^ [^^14^^]^. 

**Prediction** **of** **CTL epitope**

The CTLPred server (http://crdd.osdd.net/raghava/ ctlpred/) was used to predict CTL peptides in breast cancer antigens. The server method is based on machine learning techniques and a quantitative matrix. This server uses a combined algorithm to improve specificity and consensus prediction methods for more sensitivity. The default cut-off score performed for the prediction^[^^15^^]^. A PAComplex web server (http://pacomplex.life.nctu.edu.tw) was employed to investigate and visualize both TCR-peptide/peptide-MHC interfaces^[^^16^^]^.

**Prediction of** **MHC-I and MHC-II** **epitopes**

The following servers were utilized to predict MHC-I and MHC-II epitopes. The Rankpep server at http://imed.med.ucm.es/Tools/rankpep.html uses position-specific scoring matrices to predict MHC-I and MHC-II epitopes. The binding threshold for this server was 2-3% for MHC-I and 4-66% for MHC-II^[^^17^^]^. The second server used for MHC-I was SYFPEITHI (www.syfpeithi.de), which is a database for MHC ligands and epitope motifs and contains a collection of MHC-I ligands and peptide motifs of humans and other species^[^^18^^]^. However, MHCPred server (http://www. ddg-pharmfac.net/mhcpred/MHCPred/) was the second server used for MHC-II prediction. This server calculates IC_50_ by the QSAR stimulation method for each epitope, representing the binding affinity of the epitope to MHC-II^[^^19^^]^. Another server employed for predicting MHC-II was MHC2pred at http://crdd.osdd. net/raghava/mhc2pred/. MHC2pred server uses the vector machine method to predict MHC-II alleles and is an appropriate tool in drug design, cancer immunology, and immunotherapy, so forth^[^^20^^]^. At the end, the IEDB server (https://tools.iedb.org/mhci/) was used for both epitopes. The prediction was performed using IEDB default recommended method^[^^19^^,^^21^^]^. 

**Prediction of B** **-** **cell epitopes**

To predict B-cell epitopes, we applied three servers. First, ABCpred (http://crdd.osdd.net/raghava// abcpred/) server, which uses the ANN method, is based on the use of fixed length patterns. This server had 65.93% accuracy in the default threshold of 0.51^[^^22^^,^^23^^]^. The second server, BCpred (http://crdd.osdd.net/ raghava/ bcepred), was applied to predict B-cell epitopes. Finally, BepiPred server (http://www.cbs.dtu. dk/services/BepiPred/) was employed for predicting B-cell epitopes. This server uses a propensity scale method and combines Hidden Markov Model to predict the binding of B-cell peptides and thresholds; the score of this server is 0.35^[^^24^^]^.

**Designing recombinant vaccine sequence**

First, a pool of sequences was extracted from the above-mentioned servers to be used for vaccine design. In the second step, epitopes with high score were selected. These epitopes possess high probability for inducing the specific immune responses and considered for efficient multi-epitope vaccine. Finally, the selected segment joined together by appropriate linkers. Linker sequences were chosen based on the linkers reported in the multi-epitope peptide vaccines in the linker databases and published literature. Linkers increase the representation and proper separation of the epitopes. On the other hand, glycine-rich linkers, such as GSGSGS, can improve the flexibility and solubility. GSGSGS and AAYKK sequences were selected to join the final vaccine epitopes, and two TLR4 agonists, including RPFB and RPFE, were linked to each end of the vaccine construct by the EAAAK linker.

**Allergenicity prediction of the multi-epitope vaccine**

The following servers were selected to predict allergenicity. AlgPred web server (https://webs.iiitd. edu.in/raghava/algpred2/batch.html) predict allergenic and non-allergenic protein from their primary sequence. Machine learning models like Random Forest based on amino-acid composition and hybrid approach (RF+BLAST+MERCI) have been implemented in this server^[^^25^^]^. AllerTOP (https://www. ddg-pharmfac.net/AllerTOP/) is the second server used for allergenicity prediction. AllerTOP works based on auto cross-covariance transformation of protein sequences into uniform equal length vectors^[^^26^^]^. Another server, AllergenFP (https://www.ddg-pharmfac.net/AllergenFP/), predict allergenicity between non-allergens and allergens. This server uses a prediction method based on the novel descriptive fingerprint^[^^27^^]^.

**Antigenicity prediction of the multi-epitope vaccine**

The first server used was VaxiJen v2.0 (https://www.ddg-pharmfac.net/vaxijen/VaxiJen/ VaxiJen.html), which is based on auto cross-covariance. For predicting the protective antigens, we used principal chemical properties of proteins. The accuracy of this server ranges from 70% to 89% according to the organism^[^^28^^]^. ANTIGENpro was the second server for antigenicity prediction. The server uses machine learning algorithms to predict the results obtained by analyzing protein microarray data (http://scratch.proteomics.ics.uci.edu/)^[^^29^^]^. 

**Solubility and physiochemical properties of the multi-epitope vaccine**

SOLpro server (https://scratch.proteomics.ics. uci.edu/) uses a two-stage SVM architecture to calculate solubility predictions^[^^30^^]^. The ProtParam server (http://web.expasy.org/protparam/) was employed to compute various physical and chemical parameters for recombinant vaccine sequence^[^^31^^]^. 

**Prediction of the secondary structure**

Online web server PSIPRED (http://bioinf.cs.ucl. ac.uk/psipred/) used for protein secondary structure prediction. PSIPRED is capable of achieving a Q3 score of 81.6%. For the secondary structure prediction, this server uses two feed forward neural networks based on PSI-BLAST (Position-Specific Iterated BLAST) output ^[^^32^^]^.

**Prediction of the tertiary structure**

We used the RaptorX server (http://raptorx.uchicago. edu/) to predict the 3D structure of the novel vaccine. This server is powered by deep learning and is appropriate for protein sequences without close homologs in the PDB. It also uses confidence scores to indicate the quality of a predicted 3D model. RaptorX server possesses appropriate confidence scores from the global distance test in the absolute global quality, and this confidence score makes this server a powerful tool for tertiary structure prediction. ^[^^33^^]^.

**Refinement of the 3D structure**

Galaxy Refine server (http://galaxy.seoklab.org/) was used to improve the 3D structure of the recombinant vaccine. The method of server for relaxing the structure is the CASP10-based refining method; therefore, it is one of the most appropriate servers for enhancing the local structural quality^[^^34^^]^.

**Validation of the 3D structure**

The following web servers were used to validate and evaluate the recombinant construction. ProSA-web server (https://prosa.services.came.sbg.ac.at/prosa.php) scores the overall quality of a specific input structure and determines possible errors in the predicted structure. This server depicts an overall quality score plot that helps to realize the correctness of our prediction^[^^35^^]^. ERRAT server (http://services.mbi.ucla. edu/ERRAT/) was the next server exploited for model validation^[^^36^^]^. RAMPAGE server https://warwick.ac. uk/fac/sci/moac/people/students/peter_cock/python/ramachandran/other/ was employed for Ramachandran plot analysis^[^^37^^]^.

**Molecular docking**

The CASTp server at http://sts.bioe.uic.edu/ castp/was selected to predict the TLR-4 receptor cavities or binding pocket. The main goal of CASTp server is providing comprehensive and detailed quantitative characterization of topographic characteristics of proteins^[^^38^^]^. Molecular docking of the recombinant vaccine and TLR4 (PDB ID: 4G8A) was performed using ZDOCK server (http://zdock. umassmed.edu/). ZDOCK searches and calculates docking in all possible binding modes in rotation and translation^[^^39^^]^. 

**Molecular dynamic simulation**

The iMODS server (http://imods.chaconlab.org/) was utilized for MD study. The structural dynamics of recombinant vaccine complex were checked by using this tool due to its very rapid and efficient evaluation than other MD simulation servers^[^^40^^]^. The iMODS server provides the elastic network, covariance map, Bfactor (mobility profiles), variance, eigenvalues, and values of deformability. The server is also a simple and fast tool for measuring and determining the recombinant protein flexibility. This server with NAM explains the collective motion of proteins in the internal coordinates^[^^41^^,^^42^^]^.

***In silico*** **cloning and codon optimization**

After performing vaccine structure design, the SMS server (https://www.bioinformatics.org/sms2/rev_trans. html) was exploited for reverse translation, and the JCAT server (https://www.jcat.de/Start.jsp) for both codon optimization and quantitative codon analysis. JCAT server calculates essential parameters for cloning, including codon adaptation index and GC content^[^^43^^]^. The mentioned parameters play crucial roles in achieving a high quantity and quality expression of multi-epitope vaccine in the *E. coli* host. Ultimately, restriction enzyme sites, called *Bam*HI and *Hind*III, to both terminals of the recombinant construction were used to clone DNA sequences in the pET-28b (+) vector. 

## RESULTS

**Prediction of T-cell epitopes**

CTLpred and PAComplex servers predicted high scoring epitopes. Selected epitopes with the highest binding affinity score are shown in Table 1. MHC-I epitopes were tested using Rankpep, SYFPEITHI, and IEDB servers. The effective epitopes on human major histocompatibility complex Class-I alleles (HLA-A0102 and HLA-B0702) were predicted, as well. Thereafter, the highest-ranked epitopes with overlapping areas were selected (Tables 2, 3, and 4). Also, the effective epitopes on human major histocompatibility complex Class-II alleles (HLA-DRB) were identified. Next, the sequence of epitopes with overlapping regions was selected as the final MHC-II epitopes. The selected epitopes are shown in Tables 5, 6, and 7.

**Table 1:** Prediction of CTL epitopes from antigens by two different servers

**Antigen**	**Servers**	**Start position**	**Sequence**	**Score**
CEA				Score (ANN/SVM)
	CTLpred	216	ETQNPVSAR	1.000
		605	YLSGANLNL	1.000
		87	QQATPGPAY	0.990
			
				Best joint Z-value in the region
	PAComplex	554	RTLTLFNV	4.96
		376	RTLTLLSV	4.4
				
MTDH				Score (ANN/SVM)
	CTLpred	1122	NQYKTEAAS	1.000
		45	VPSSTEKNA	0.990
		126	PAPGSTAPP	0.990
			
				Best joint Z-value in the region
	PAComplex	1050	SNLQFNSS	4.64
				
MUC-1				Score (ANN/SVM)
	CTLpred	1122	NQYKTEAAS	1.000
		45	VPSSTEKNA	0.990
		126	PAPGSTAPP	0.990
			
				Best joint Z-value in the region
	PAComplex	1050	SNLQFNSS	4.64
		1207	SEYPTYHT	4.17
		1119	TQFNQYKT	4.01

**B-cell epitope prediction**

B-cell epitopes are a major player in humoral responses; therefore, ABCpred, BCpred, and BepiPred servers were used to identify potential B-cell epitopes. Table 8 shows the linear B-cell epitopes selected from the antigens.

**Vaccine construct**

**Fig. 2:**
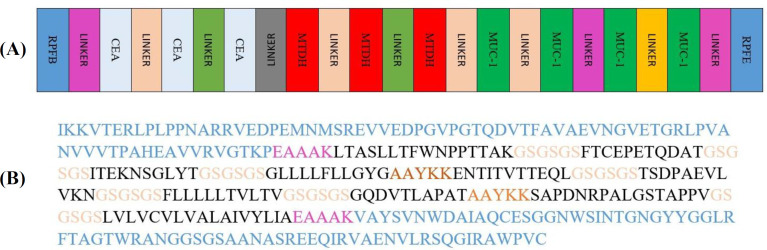
(A) schematic illustration of the final multi-epitope vaccine construct. (B) The chimera sequence of final novel vaccine construct

Based on the highly ranked and overlapped MHC-I, MHC-II, CTL, and B-cell epitopes (Table 9), of the three antigens selected for breast cancer, 10 sections were selected as final areas. The ultimate epitopes were joined by GSGSGS and AAYKK linkers. Finally,the EAAAK linker was added as RpfE and RpfB adjuvants to the beginning and end of the vaccine construct (Fig. 2).

**Table 2:** Prediction of MHC-I epitopes from CEA by three different servers

**Servers**	**Start position**	**Sequences**	**Best ranked epitope in the region**
Rankpep			Score^a^
	467	ITEKNSGLY	26.094
	205	VTRNDTASY	17.906
	241	TISPLNTSY	16.692
	645	ITPNNNGTY	16.283
		
			Score^b^
SYFPEITHI	467	ITEKNSGLY	30
	168	EPETQDAT	24
	346	EPEIQNTTY	24
	524	EPEAQNTTY	24
		
			Percentile rank^c^
IEBD	40	NRSDPVTLDVLY	0.05
	5	YLSGANLNL	0.05
	38	SANRSDPVTLDVLY	0.05
	8	SPRLQLSNGNRTL	0.05

^a^High, ^b^high Log, and ^c^low percentile¼good binder

**Table 3:** Prediction of MHC-I epitopes from MTDH by three different servers

**Server**	**Start position**	**Sequences**	**Best ranked epitope in the region**
Rankpep			Score^a^
	517	STEPSVILS	15.738
	211	LTDSGSLDS	12.5
	495	TSDPAEVLV	11.929
	356	VSQSTTSDY	11.762
			
SYFPEITHI			Score^b^
	211	LTDSGSLDS	21
	61	LLLLFLLGY	20
	356	VSQSTTSDY	20
	517	STEPSVILS	18
			
IEBD			Percentile rank^c^
	1	TSDYQWDVSRNQPY	0.04
	47	YPGWVILVGTGALG	0.52

^ a^High, ^b^high log, and ^c^low percentile¼good binder

**Allergenicity and antigenicity evaluation**

The results of the servers mentioned above showed that the recombinant vaccine is not allergenic. Vaxigen and ANTIGENpro servers demonstrated the probability of recombinant construction antigenicity prediction of 0.5866 and 0.93, respectively; the scores indicate that our recombinant construction can produce an effective immune response.

**Physicochemical characteristics** **and solubility evaluation of the target vaccine**

Theoretical pI and molecular weight of the designed protein were calculated as 5.43 and 33.9 kDa, respectively. The pI value implies that the designed vaccine is acidic. The overall numbers of positively and negatively charged amino acid residues were 25 and 28, respectively. The instability index was calculated to be 27.56. This result classifies the vaccine construct as stable. The aliphatic index was computed to be 80.42 by the SOLpro server. This alphabetical index explains that the protein structure of vaccine is stable in a varied range of temperatures. In addition, the GRAVY amount of the multi-epitope construct calculated as -0.073. The negative scores of GRAVY index display that the construct is hydrophilic and possess better interaction with surrounding water molecules. Vaccine solubility was predicted by SOLpro server. The probability of vaccine solubility was calculated to be 0.86. This result explains that the vaccine protein is possibly soluble, and when it is overexpressed in the *E. coli* host. 

**Table 4:** Prediction of MHC-I epitopes from MUC-1 by three different servers

**Server**	**Start position**	**Sequences**	**Best ranked epitope in the region**
Rankpep			Score^a^
	940	SAPDNRPAL	22.273
	971	TLVHNGTSA	10.903
	1171	VALAIVYLI	9.398
	1186	CRRKNYGQL	9.074
			
SYFPEITHI			Score^b^
	940	SAPDN PAL	29
	48	STEKNAVSM	25
	1186	CRRKNYGQL	20
	120	SAPDNKPAP	19
			
IEBD			Percentile rank^c^
	35	NSSLEDPSTDYY	0.04
	5	PMSEYPTYHTHGRY	0.31
	17	RYVPPSSTDRSPY	0.4

^ a^High, ^b^high log, and ^c^low percentile¼good binder

**Table 5:** Prediction of MHC-II epitopes from CEA by four different servers

**Server**	**Start position**	**Sequences**	**Rank**
Rankpep			Score^a^
	15	WQRLLLTAS	12.274
	81	GYVIGTQQA	9.862
	546	RLQLSNGNR	9.103
	453	GNIQQHTQE	6.885
		
MHCpred			Predicted IC_50_ value (nM)^b^
	691	IMIGVLVGV	16.18
	519	VAFTCEPEA	22.23
	341	VALTCEPEI	26.67
	589	VLYGPDTPI	29.51
MHC2pred			Score^c^
	533	LWWVNGQSL	1.587
	688	TVGIMIGVL	1.453
	75	GNRQIIGYV	1.425
	467	ITEKNSGLY	1.424
			
IEDB			Percentile rank^d^
	12	CIPWQRLLLTASLLT	0.91
	27	ATVGIMIGVLVGVAL	4.20
	37	GREIIYPNASLLIQN	11.00

^a^High score¼good binder;^ b^low predicted IC_50_ value¼good binder; ^c^high score = good binder; ^d^low percentile rank = good binder

**Table 6.** Prediction of MHC-II epitopes from MTDH by four different servers

**Server**	**Start position**	**Sequences**	**Rank**
Rankpep			Score^a^
	79	RKKRRSPPR	11.409
	25	LSVGLGFLR	10.851
	119	RKKLSEKPK	9.069
	343	RSIFSGIGS	8.897
	137	EGEAVRTPQ	8.408
			
MHCpred			Predicted IC50 value (nM)^b^
	374	YIDDEWSGL	1.11
	6	WQDELAQQA	1.38
	233	EQLTTASFP	1.82
	144	PQSVTAKQP	2.97
			
MHC2pred			Score^c^
	278	NLTVNGGGW	1.870
	218	DSTIPGIEN	1.717
	516	TSTEPSVIL	1.497
	283	GGGWNEKSV	1.464
			
IEDB			Percentile rank^d^
	41	DDLALLKNLRSEEQK	1.80
	2	LLLFLLGYGWAAACA	2.40
	9	NSQPIKTLPPATSTE	2.80

^a^High score¼good binder; ^b^Low predicted IC_50_ value¼good binder; ^c^high score = good binder; ^d^low percentile rank = good binder

**Table 7:** Prediction of MHC-II epitopes from MUC-1 by ,four different servers

**Server**	**Start position**	**Sequences**	**Rank**
Rankpep			Score^a^
	11	LLLLLTVLT	10.731
	1040	VSFFFLSFH	8.514
	1156	VPGWGIALL	6.456
	1105	LAFREGTIN	6.027
			
MHCpred			Predicted IC50 value (nM)^b^
	102	PVTRPALGS	0.98
	10	FLLLLLTVL	1.09
	82	PATEPASGS	2.01
	75	GQDVTLAPA	2.74
			
MHC2pred			Score^c^
	1083	QGGFLGLSN	1.716
	70	SSTTQGQDV	1.545
	1079	QIYKQGGFL	1.506
	14	LLTVLTVVT	1.491
			
IEDB			Percentile rank^d^
	29	VLVALAIVYLIALAV	0.10
	8	PFFLLLLLTVLTVVT	0.16

^a^High score¼good binder;^ b^low predicted IC_50_ value¼good binder; ^c^high score = good binder; ^d^low percentile rank = good binder

**Table 8:** Prediction of B cell epitopes by BCpred, ABCpred, and BepiPred servers

**Proteins**	**Start–end position**	**Sequence**	**Best ranked epitope** **in the region**
CEA			Score^a^
	175	GREIIYPNASLLIQNI	0.96
	247	PETQDATYLWWVNNQS	0.93
	676	ISPPDSSYLSGANLNL	0.91
	395	TVYAEPPKPFITSNNS	0.91
	424	EPEIQNTTYLWWVNNQ	0.89
	55	ESHMSAPIENSXGNCE	0.87
			
MTDH			Score^a^
	366	AWSQDTGDANTNGKDW	0.94
	403	QSTTSDYQWDVSRNQP	0.93
	197	PPEIDKKNEKSKKNKK	0.93
	428	NGLSSADPNSDWNAPA	0.92
	48	ARSWQDELAQQAEEGS	0.88
			
MUC-1			Score^a^
	1236	HPMSEYPTYHTHGRYV	0.95
	1008	GTSARATTTPASKSTP	0.94
	948	HGVTSAPDTRPAPGST	0.92
	15	PPAHDVTSAPDNKPAP	0.85
	91	SVLSSHSPGSGSSTTQ	0.81
	109	DVTLAPATEPASGSAA	0.64

^ a^High score = good binder

**Table 9:** The final immunodominant epitope sequences selected from CEA, MTDH, and MUC-1 antigens

**Protein**	**Start position**	**End position**	**Epitopes**
CEA	20	35	LTASLLTFWNPPTTAK
	165	175	FTCEPETQDAT
	467	476	ITEKNSGLYT
			
MTDH	60	70	GLLLLFLLGYG
	225	235	ENTITVTTEQL
	495	505	TSDPAEVLVKN
			
MUC-1	10	20	FLLLLLTVLTV
	75	85	GQDVTLAPAT
	940	955	SAPDNRPALGSTAPPV
	1164	1180	LVLVCVLVALAIVYLIA

**Homology modeling**

**Fig. 3:**
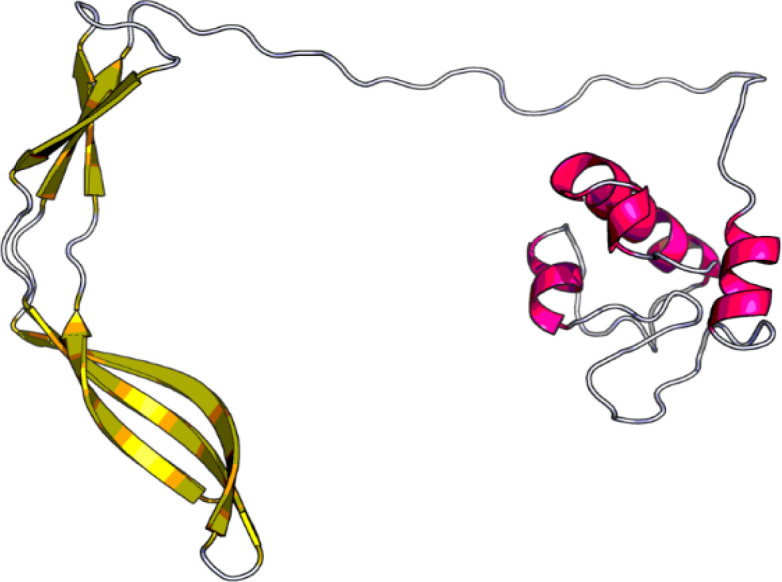
The 3D structure of designed vaccine generated by Raptor X server. This server uses deep learning and provides a powerful tool for 3D structure prediction of proteins that do have not any close homologs in the PDB

We used the RaptorX server for the homology modeling. Raptor X server calculated *p* value, at 6.64e-10. Also, overall uGDT was equal to 144. These values display that 3D modeled structure is acceptable (Fig. 3).

**secondary structure Prediction**

**Fig. 4:**
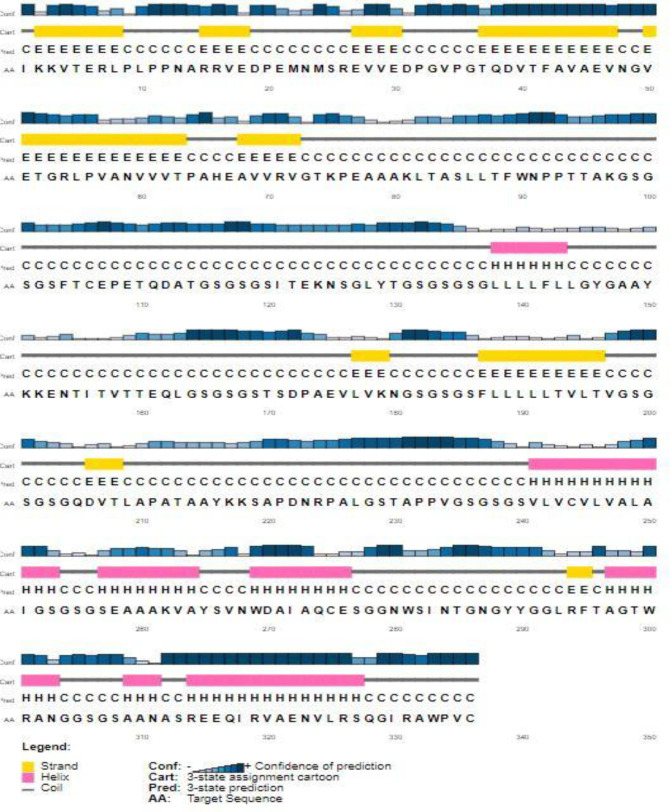
PSIPRED graphical output from secondary structure prediction of the novel vaccine. The blue bars show the confidence of prediction

The secondary structure of the designed vaccine contains 26.19% extended strand, 19.64% alpha helix, and 54.17% coil structural elements, as predicted by the PSIPRED server (Fig. 4).

**Refinement of 3D structure**

Five 3D refined models were introduced by GalaxyRefine server, and all models entered to model validation step. According to potential errors, the best model was selected based on the z-score and the overall quality factor.

**Refined tertiary structure validation**

**Fig. 5:**
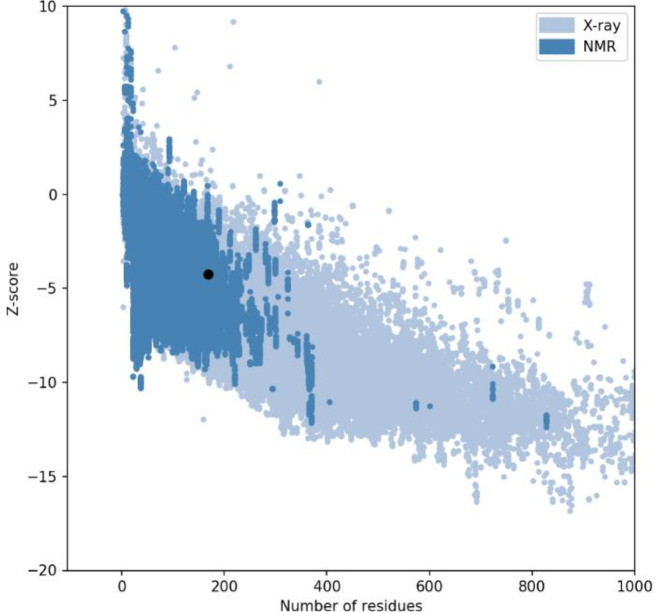
The z-score plot for 3D structure of constructs. The z-score of a model stability processes is -5.3, which is in the range of native protein conformation

**Fig. 6:**
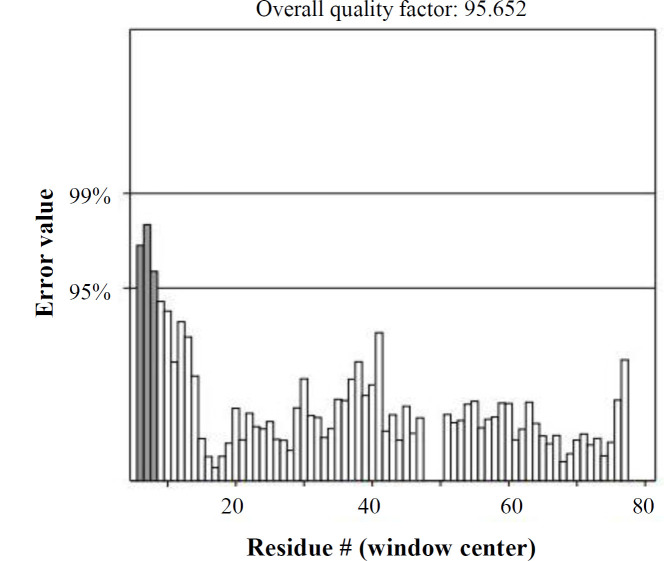
Errat plot for the designed multi-epitope vaccine model. The overall quality factor plot (ERRAT) of the model stability was calculated at 96.154%. Gray bars demonstrate the error region between 95% and 99%, and white bars indicate the region with a lower error rate for protein folding

**Fig. 7:**
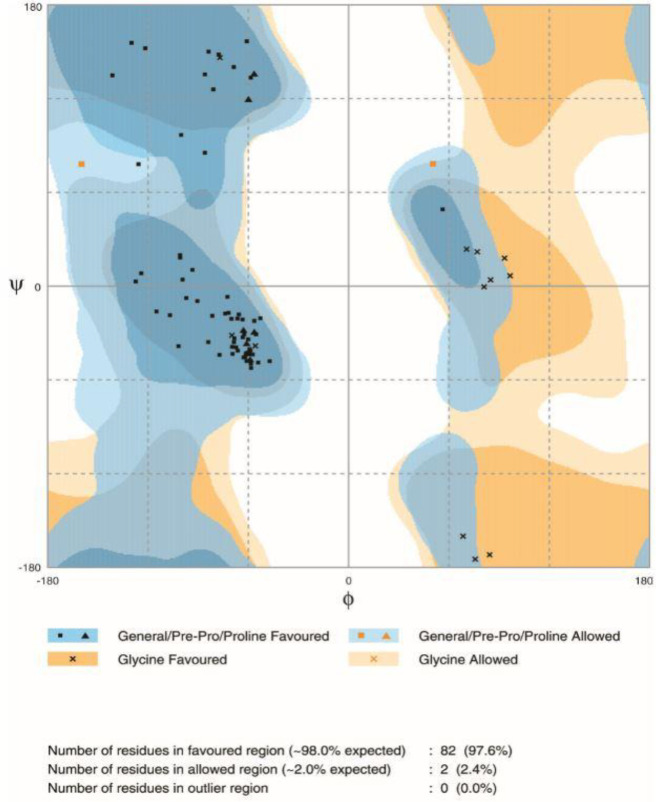
Calculation of model stability based on Ramachandran plot

ProSA-web, ERRAT, and RAMPAGE servers were utilized for calculating potential errors in the initial best model. ProSA-web computes z-score as -5.2 for the refined model. This calculated score is in the range of native proteins with similar size scores (Fig. 5). The ERRAT server was employed for the quality assessment of the modeled construct. ERRAT outputs indicate that the quality factor of the predicted 3D model was 95.65% (Fig. 6). The RAMPAGE server analysis revealed that residues in the outlier region were 1 (0.6%), residues in the allowed region were 1 (0.6 %), and the number of residues in favored region was 164 (98.8%), as depicted in Figure 7.

**Molecular docking of subunit vaccine with TLR-4**

**Fig. 8.**
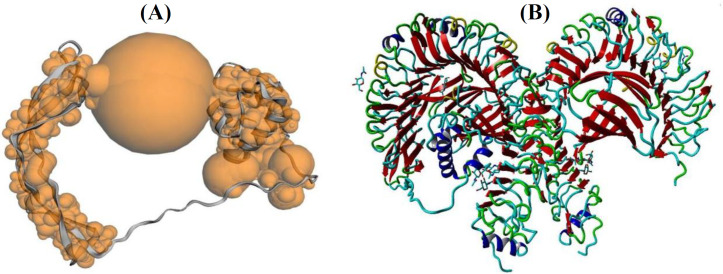
Docking model of recombinant vaccine and TLR4 complex achieved by CASTp and ZDOCK servers. (A) Hydrophobic interaction and protein binding site on protein surface determined by CASTp server; (B) recombinant vaccine-mediated targeted docking against TLR4 performed by ZDOCK server at the predicted binding pocket

Hydrophobic interaction and protein binding site on protein surface were determined by CASTp server. The server identified a possible binding to the TLR4 receptor in the binding pocket in amino acids 32-616. 134.4 Å^2 ^was calculated as the size of the molecular surface pocket, and the molecular surface volume was calculated as 188.96 Å^3^. The ZDOCK server used a 

recombinant vaccine against TLR4 to predict the binding pocket (Fig. 8). This server generates top 10 models based on all possible binding modes in the translational and rotational space between the two proteins and evaluates each pose using an energy-based scoring function. The best docking model was then selected according to reaction and action sites and visualized by using YASARA software.

**Molecular dynamic simulation of recombinant vaccine molecule**

**Fig. 9:**
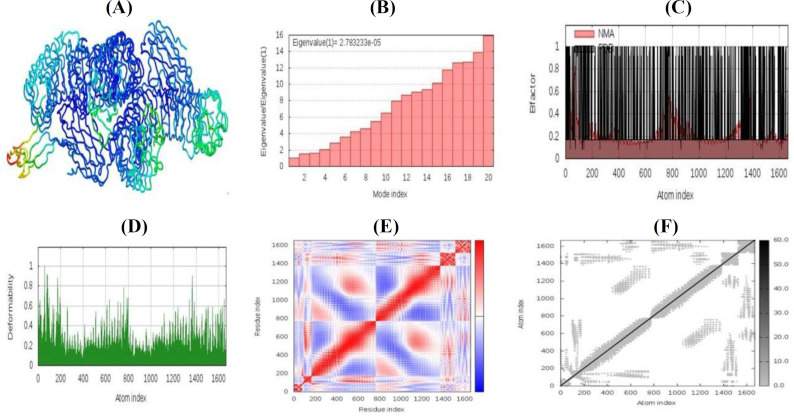
A schematic displaying the findings of MD simulation study of TLR-4 and recombinant vaccine docked complex. (A) NMA mobility; (B) deformability; (C) B-factor; (D) eigenvalues; (E) covariance; (F) elastic network analysis

The best rating collection between TLR-4 and recombinant vaccine molecules was selected for the analysis through NMA. Figure 9A exhibits the MD simulation and NMA of docked complex. The deformability diagrams of the docked complex and peaks in the diagrams indicate the areas of the designed vaccine with deformability (Fig. 9B). The B-factor diagram of the complexes provides visualization and simple understanding of the comparison among the PDB field and the NMA of the docked complex (Fig. 9C). Figure 8D shows the dedicated values of the docked complex. TLR4 and recombinant vaccine docked complex produced eigenvalue of 2.783233e-05.

As depicted in Figure 9E the covariance map of the docked complex reveals the uncorrelated motion by white, anticorrelated motion by blue, and correlated motion between a pair of residues by red colors (Fig. 9C). The elastic network diagram of the docked complex distinguishes pairs of atoms associated with helixes and shows the hardness of connection between the atoms (Fig. 9F).

***In Silico*** **optimization for molecular cloning**

The designed novel vaccine was translated into DNA sequence by using the SMS web server. In the next step, to obtain a maximal protein expression in *E. coli*, JCAT web server was applied for codon optimization. Following the optimization of the nucleotide sequence, codon adaptation index with a score of 1.0 and a GC content of 53.17% was achieved. Based on the obtained data, a high-level expression of the designed construct would be expected in *E. coli* K12strain.

## DISCUSSION

One of the severe forms of breast cancer is TNBC. This subtype is known for poor prognosis and difficult treatment. Also, this type of breast cancer displays short overall survival, strong invasive nature, and a high degree of malignancy. The disease is always

recognized in advanced phases with extensive metastasis^[^^44^^,^^45^^]^. These characteristics of TNBC, along with the limited therapy options, have made immunotherapy an attractive option for TNBC treatment.

The immune system plays an important role in cancer management and recognizes malignant cells that display tumor antigens through MHC complexes.^[^^47^^]^ Multi-epitope vaccines are designed to induce or intensify a population of T lymphocytes and can recognize and eradicate cancer cells. Therefore, choosing appropriate antigens is important for vaccine design. Previous studies and trials have demonstrated that tumor antigens, Her-2, CEA, and MUC-1, are safe and can induce immune system responses^[^^46^^]^. As the present study aimed to design a novel vaccine against TNBC, we selected MTDH in addition to CEA and MUC-1 antigens. 

Since using epitopes, which activate B-cell and CTL, as well as MHC-I and MHC-II molecules, help us to fight TNBC tumor cells, we applied *in silico* tools to find the most immunogenic sequence from TNBC antigens that are involved in tumor progression and metastasis. 

Besides the importance of antigen selection in cancer vaccine design, choosing appropriate adjuvant plays a crucial role in recombinant vaccine efficiency. Researchers have introduced different adjuvants, such as aluminum salt, Montanide, and TLR agonists, in various investigations^ [^^48^^-^^52^^]^. In this research, to create a powerful immune response, we used TLR4 agonist, which has powerful stimulatory characteristics, to improve the immunogenicity of the novel designed construct. TLR4 possesses unique properties between other TLRs and stimulates both cellular and humoral immune system at the same time^[^^53^^]^. 

After the selection of antigens and adjuvants, appropriate linkers are important to produce a protein with optimal performance. Herein, we used several linkers, namely GSGSGS, EAAAK, and AYYKK. GSGSGS flexible linkers do not alter the properties of the peptide epitopes and are applied to attach functional domains. EAAAK rigid linkers have been selected to ensure stability in the vaccine structure at a fixed distance between the epitopes and the adjuvants^[^^54^^,^^55^^]^. AAYKK cleavable linker also used in this construct. This linker is cleaved by cathepsin B and makes double lysine (KK) site and AAY motif^[^^56^^]^. AAY motif after linker cleavage is suitable for binding to TAP transporter, which has a significant role in epitope offering to the immune system^[^^57^^]^.

Physicochemical, structural and immunological features of the designed recombinant construction were evaluated by several immunoinformatics servers. These results indicated the instability index of 34.63, and it categorized this protein as resistant. Immunological analysis showed that the designed vaccine is not allergen. Predicting the secondary structure has a crucial function in the performance and 3D model of the recombinant vaccine structure. PSIPRED server is a very useful and very accurate approach used in this study to analyze the secondary structure of the novel vaccine. The 3D structure of the novel vaccine affects its biological function^[^^58^^]^. In the present study, the RaptorX server was selected to model the 3D structure. ProSA-web, RAMPAGE, and ERRAT servers were chosen to identify possible errors and to improve the overall quality of the predicted 3D model. Then the final model was used to evaluate docking with TLR4. The two servers were utilized for the docking study. CastP server is based on theoretical and algorithmic results of computational geometry and calculates topological possibilities for protein interactions. The Z-DOCK server evaluates any protein using an energy-based scoring function. 

The designed vaccine in complex with TLR-4 was subjected to MD simulation. The MD simulation study revealed that the TLR-4-TNBC vaccine docked complex is needed to be stable with a strong eigenvalue. On the other hand, this complex needs quite stability in the biological environment, because of less chance of deformation. As shown in Figure 9E, the complex possesses a good number of amino acids that were in the correlated motion.

Codon optimization was performed to obtain the high expression level of novel vaccine in *E. coli*^[^^59^^]^. The results indicated that the percentage of solubility score for designed construct is 0.86%, which demonstrates the high probability for overexpressing multi-epitope vaccine in a soluble form after overexpression in *E. coli*.

In summary, the present study was carried out to design a recombinant multi-epitope vaccine construct for cancer immunotherapy. The designed multi-epitope vaccine includes B-cell, MHC-I, MHC-II, and CTL epitopes that were fused with suitable linkers to stimulate both humoral and cellular immunity. Following bioinformatics assessments, the designed vaccine showed a potential therapeutic feature against TNBC. However, further *in vitro* and in* vivo* investigations are required to confirm its biological activity.

## DECLARATIONS

### Ethical statement

Not applicable.

### Data availability

The numerical model simulations upon which this study is based are too large to archive or transfer. Instead, we provide all the information needed to replicate the simulations.

### Author contributions

PH, and HD conceived the presented idea, developed the theory and performed the computations. VG, and SA were responsible for the study and identification of overexpressed antigen in TNBC, verified the analytical methods and supervised the findings of this work. All authors discussed the results and contributed to the final manuscript.

### Conflict of interest

None declared.

### Funding/support

This research did not receive any specific grant from funding agencies in the public, commercial, or not-for-profit sectors.

## References

[1] Esteva FJ, Hubbard-Lucey VM, Tang J, Pusztai L (2019). Immunotherapy and targeted therapy combinations in metastatic breast cancer. The lancet oncology.

[2] Katz H, Alsharedi M (2018). Immunotherapy in triple-negative breast cancer. Medical oncology.

[3] Crown J, O'shaughnessy J, Gullo G (2012). Emerging targeted therapies in triple-negative breast cancer. Annals of oncology.

[4] O’Neill S, Porter RK, McNamee N, Martinez VG, O'Driscoll L (2019). 2-Deoxy-D-Glucose inhibits aggressive triple-negative breast cancer cells by targeting glycolysis and the cancer stem cell phenotype. Scientific reports.

[5] de Paula Peres L, da Luz FAC, dos Anjos Pultz B, Brígido AC, Agenor de Araújo R, Ricardo Goulart L, Barbosa Silva MJ (2015). Peptide vaccines in breast cancer: The immunological basis for clinical response. Biotechnology advances.

[6] Pol JG, Bridle BW, Lichty BD (2020). Detection of Tumor Antigen-Specific T-Cell Responses After Oncolytic Vaccination. Methods in molecular biology.

[7] Shahid F, Ashfaq UA, Javaid A, Khalid H (2020). Immunoinformatics guided rational design of a next generation multi epitope based peptide (MEBP) vaccine by exploring Zika virus proteome. Infection, genetics and evolution.

[8] Fikes JD, Sette A (2003). Design of multi-epitope, analogue-based cancer vaccines. Expert opinion on biological therapy.

[9] Bayraktar S, Batoo S, Okuno S, Gluke S (2019). Immunotherapy in breast cancer. Journal of carcinogenesis.

[10] Brown DM, Ruoslahti E (2004). Metadherin, a cell surface protein in breast tumors that mediates lung metastasis. Cancer cell.

[11] Criscitiello C (2012). Tumor-associated antigens in breast cancer. Breast care.

[12] Vermaelen K (2019). Strategies to Improve Cancer Vaccine Efficacy. Frontiers in immunology.

[13] Bairoch A, Apweiler R, Wu CH, Barker WC, Boeckmann B, Ferro S, Gasteiger E, Huang H, Lopez R, Magrane M, Martin MJ, Natale DA, O'Donovan C, Redaschi N, L Yeh L-S (2005). The universal protein resource (UniProt). Nucleic acids research.

[14] Park BS, Song DH, Kim HM, Choi B-S, Lee H, Lee J-O (2009). The structural basis of lipopolysaccharide recognition by the TLR4–MD-2 complex. Nature.

[15] Bhasin M, Raghava G (2004). Prediction of CTL epitopes using QM, SVM and ANN techniques. Vaccine.

[16] Liu I-H, Lo Y-S, Yang J-M (2011). PAComplex: a web server to infer peptide antigen families and binding models from TCR–pMHC complexes. Nucleic acids research.

[17] Reche PA, Glutting J-P, Reinherz EL (2002). Prediction of MHC class I binding peptides using profile motifs. Human immunology.

[18] Rammensee H-G, Bachmann J, Emmerich NPN, Bachor OA, Stevanović S (1999). SYFPEITHI: database for MHC ligands and peptide motifs. Immunogenetics.

[19] Guan P, Doytchinova IA, Zygouri C, Flower DR (2003). MHCPred: a server for quantitative prediction of peptide–MHC binding. Nucleic acids research.

[20] Kangueane P, Sakharkar MK (2005). T-Epitope Designer: A HLA-peptide binding prediction server. Bioinformation.

[21] Wang P, Sidney J, Dow C, Mothé B, Sette A, Peters B (2008). A systematic assessment of MHC class II peptide binding predictions and evaluation of a consensus approach. Plos computational biology.

[22] Hashemzadeh P, Ghorbanzadeh V, Otaghsara SMV (2020). Novel predicted B-cell epitopes of PSMA for development of prostate cancer vaccine. International journal of peptide research and therapeutics.

[23] EL‐Manzalawy Y, Dobbs D, Honavar V (2008). Predicting linear B‐cell epitopes using string kernels. Journal of molecular recognition.

[24] Larsen JEP, Lund O, Nielsen M (2006). Improved method for predicting linear B-cell epitopes. Immunome research.

[25] Saha S, Raghava G (2006). AlgPred: prediction of allergenic proteins and mapping of IgE epitopes. Nucleic acids research.

[26] Dimitrov I, Bangov I, Flower DR, Doytchinova I (2014). AllerTOP v 2—a server for in silico prediction of allergens. Journal of molecular modeling.

[27] Dimitrov I, Naneva L, Doytchinova I, Bangov I (2013). AllergenFP: allergenicity prediction by descriptor fingerprints. Bioinformatics.

[28] Doytchinova IA, Flower DR (2007). VaxiJen: a server for prediction of protective antigens, tumour antigens and subunit vaccines. BMC bioinformatics.

[29] Magnan CN, Zeller M, Kayala MA, Vigil A, Randall A, Felgner P-L, Baldi P (2010). High-throughput prediction of protein antigenicity using protein microarray data. Bioinformatics.

[30] Magnan CN, Randall A, Baldi P (2009). SOLpro: accurate sequence-based prediction of protein solubility. Bioinformatics.

[31] Gasteiger E, Hoogland C, Gattiker A, Duvaud S, Wilkins MR, Appel RD, Bairoch A (2005). Protein identification and analysis tools on the ExPASy server. The proteomics protocols handbook.

[32] Buchan DW, Jones DT (2019). The PSIPRED protein analysis workbench: 20 years on. Nucleic acids research.

[33] Peng J, Xu J (2011). RaptorX: exploiting structure information for protein alignment by statistical inference. Proteins.

[34] Heo L, Park H, Seok C (2013). GalaxyRefine: protein structure refinement driven by side-chain repacking. Nucleic acids research.

[35] Wiederstein M, Sippl MJ (2007). ProSA-web: interactive web service for the recognition of errors in three-dimensional structures of proteins. Nucleic acids research.

[36] Colovos C, Yeates TO (1993). Verification of protein structures: patterns of nonbonded atomic interactions. Protein science.

[37] Lovell SC, Davis IW, Arendall 3 WB, de Bakker PIW, Michael Word J, Prisant MG, Richardson JS, Richardson DC (2003). Structure validation by Cα geometry: Phi, psi and Cbeta deviation. Proteins.

[38] Tian W, Chen C, Lei X, Zhao J, Liang J (2018). CASTp 3. 0: computed atlas of surface topography of proteins. Nucleic acids research.

[39] Pierce BG, Hourai Y, Weng Z (2011). Accelerating protein docking in ZDOCK using an advanced 3D convolution library. Plos one.

[40] Awan FM, Obaid A, Ikram A, Janjua Ha (2017). Mutation-structure-function relationship based integrated strategy reveals the potential impact of deleterious missense mutations in autophagy related proteins on hepatocellular carcinoma (HCC): A comprehensive informatics approach. International journal of molecular sciences.

[41] Tama F, Brooks CL (2006). Symmetry, form, and shape: guiding principles for robustness in macromolecular machines. Annual review of biophysics and biomolecularstructure.

[42] López-Blanco JR, Aliaga JI, Quintana-Ortí ES, Chacon P (2014). iMODS: internal coordinates normal mode analysis server. Nucleic acids research.

[43] Grote A, Hiller K, Scheer M, Münch R, Nörtemann B, Hempel DC, Jahn D (2005). JCat: a novel tool to adapt codon usage of a target gene to its potential expression host. Nucleic acids research.

[44] Huynh M-m, Jayanthan A, Pambid MR, Los G, Dunn SE (2020). RSK2: a promising therapeutic target for the treatment of triple-negative breast cancer. Expert opinion on therapeutic targets.

[45] Li L, Zheng X, Zhou Q, Villanueva N, Nian W, Liu X (2020). Metabolomics-Based Discovery of Molecular Signatures for Triple Negative Breast Cancer in Asian Female Population. Scientific reports.

[46] Emens LA (2018). Breast cancer immunotherapy: facts and hopes. Clinical cancer research.

[47] Soria-Guerra RE, Nieto-Gomez R, Govea-Alonso DO, Rosales-Mendoza S (2015). An overview of bioinformatics tools for epitope prediction: implications on vaccine development. Journal of biomedical informatics.

[48] Childers NK, Miller KL, Tong G, Llarena JC, Greenway T, Ulrich JT, Michalek SM (2000). Adjuvant activity of monophosphoryl lipid A for nasal and oral immunization with soluble or liposome-associated antigen. Infection and immunity.

[49] Cluff CW (2009). Monophosphoryl lipid A (MPL) as an adjuvant for anti-cancer vaccines: clinical results. Advances in experimental medicine and biology.

[50] Didierlaurent AM, Morel S, Lockman L, Giannini SL, Bisteau MI, Carlsen H, Kielland A, Vosters O, Vanderheyde N, Schiavetti F, Larocque D, Van Mechelen M, Garçon N (2009). AS04, an aluminum salt-and TLR4 agonist-based adjuvant system, induces a transient localized innate immune response leading to enhanced adaptive immunity. Journal of immunology.

[51] Steinhagen F, Kinjo T, Bode C, Klinman DM (2011). TLR-based immune adjuvants. Vaccine.

[52] Shirota H, Tross D, Klinman DM (2015). CpG oligonucleotides as cancer vaccine adjuvants. Vaccine.

[53] Duthie MS, Windish HP, Fox CB, Reed SG (2011). Use of defined TLR ligands as adjuvants within human vaccines. Immunological reviews.

[54] Chen X, Zaro JL, Shen W-C (2013). Fusion protein linkers: property, design and functionality. Advanced drug delivery reviews.

[55] Reddy Chichili VP, Kumar V, Sivaraman J (2013). Linkers in the structural biology of protein–protein interactions. Protein science.

[56] Minhas V, Shrestha A, Wadhwa N, Singh R, Gupta SK (2016). Novel Sperm and Gonadotropin-releasing Hormone-based Recombinant Fusion Protein: Achievement of 100% Contraceptive Efficacy by Co-immunization of Male and Female Mice. Molecular reproduction and development.

[57] de Oliveira LMF, Morale MG, Chaves AAM, Cavalher AM, Lopes AS, de Oliveira Diniz M, Schanoski AS, Lopes de Melo R, Carlos de Souza Ferreira L, S de Oliveira ML, Demasi M, Lee Ho P (2015). Design, immune responses and anti-tumor potential of an HPV16 E6E7 multi-epitope vaccine. Plos one.

[58] Ashokan K, Pillai M (2008). In silico characterization of silk fibroin protein using computational tools and servers. Asian journal of experimental sciences.

[59] Makrides SC (1996). Strategies for achieving high-level expression of genes in Escherichia coli. Microbiological reviews.

